# Health Uninsurance in rural America: a partial equilibrium analysis

**DOI:** 10.1186/s13561-019-0234-x

**Published:** 2019-06-19

**Authors:** William Nganje, Kwame Asiam Addey

**Affiliations:** 1 Department of Agribusiness and Applied Economics, North Dakota State University, 500 Richard H Barry Hall, Fargo, ND 58108-6050 USA; 2 Center for Agricultural Policy and Trade Studies, North Dakota State University, 524 Richard H Barry Hall, Fargo, ND 58102-6050 USA

**Keywords:** Pre-existing conditions, Principal-agent model, Rural health uninsurance, Complementary log-log binomial, Spence-Mirrlees condition, D82, I11, I13

## Abstract

**Background:**

The cost of rural health continues to be high in the United States despite an overall improvement in national health insurance enrolment. Stakeholder’s perception of adverse selection remains a paramount culprit in the challenges of rural insurance markets. Risk attitude has been revealed as an alternative for measuring this phenomenon, given the 2014 prohibition law on pre-existing conditions and a subsequent repeal in 2018 accompanied by extensive debate among congress. We examine the existence of adverse selection in rural insurance markets by comparing the effects of pre-existing or chronic health conditions and risk attitudes in a Principal-Agent model.

**Results:**

Using multinomial logit and complementary log-log binomial link models in a Principal-Agent framework, our results indicate that there is adverse selection in rural health insurance markets if pre-existing conditions are considered, but risk attitudes yield contrary effects.

**Conclusions:**

The major policy implication from this study is that respondents who have pre-existing/chronic conditions tend to patronise health insurance with a higher probability than other counterparts and therefore insurers are likely to incur losses given the law on pre-existing conditions as private information. The 2018 law on the exclusion of individuals with pre-existing conditions may be beneficial to the insurance companies at the expense of the populace. Hence, we suggest that market incentive-based programs should be encouraged to minimize rural health uninsurance.

## Introduction

Healthcare policies might have unintended consequences including market failure. The challenge is to understand what the specific consequences would be and how to resolve them. Pope [23] noted that “of the 650 counties that have only a single insurer offering plans on their exchange, 70% percent are rural”. In general, positive strides have been made by the United States in improving health insurance coverage from 86.7% in 2013 to 90.9% in 2015 [3]. Despite this improvement, rural America bears most of the health uninsured. The Minnesota Department of Health [19] revealed a 12.9% uninsurance rate for rural areas compared to 10.8% for urban areas. It has been well documented that residents of rural areas encounter more restricted access to health insurance than their urban counterparts [21, 32]. The theoretical canker of market failure in insurance markets have long been established as a cause for uninsurance or underinsurance [25]. Mainly pivoted on asymmetry of information, the two major sources of market failure are adverse selection and moral hazard. Boone [5] stated the existence of a conspicuous assertion that the basic insurance suffers from adverse selection but not moral hazard.

Given the importance of adverse selection and rural health outcomes, this study examines adverse selection in rural U.S. health insurance^1^ markets with specific focus on the behavioural characteristics of the agent. The effects of these characteristics are then compared to the effect of having a pre-existing health condition on the choice of health insurance. Since its nascent study in the 1970s, there have been several enquiries into the impact of adverse selection in health insurance markets. For most of these studies, the expected risk costs are measured based on chronic or pre-existing conditions of the customer. The basic theoretical underpinning is that, if consumers have private information about their risk of suffering a loss, there will be a positive correlation between risk and level of insurance coverage [6].

One of the most contentious contemporary issues in the US is the debate on pre-existing conditions. Pre-existing conditions were used to deny or discriminate prices at the insurance marketplaces until 2014. Posthumously, the prohibition of health insurance issuers from refusing coverage based on patients’ medical history makes pre-existing or chronic conditions alone untenable for the assessment of adverse selection behaviour. To support this assertion, the Department of Health and Human Services [30] suggested that people with pre-existing conditions were likely to have seen progress after the market reform with the Affordable Care Act. The study found that between 2010 and 2014, the uninsured rate fell by 27% while from 2014 to 2016, it fell by 22%. However, this legislation on pre-existing conditions was again repealed in 2018 implying that people with pre-existing conditions are no longer protected under the Affordable Care Act. The law on pre-existing conditions implies that insurance companies can’t refuse to cover an individual or charge them more just because they have a “pre-existing condition” — that is, a health problem the individual had before the date that new health coverage starts. These rules went into effect for plan years beginning on or after January 1, 2014. However, this rule was repealed in 2018, implying that health insurance companies can discriminate based on the existence of pre-existing conditions. Some sections of the country feel the government is being too harsh on people with pre-existing conditions while this may be an answer to the age-long cry of health insurance providers. The government believes this action will lead to lower premiums.

This debate may persist for a considerable length of time and is even more important for rural dwellers. In general, health insurance in the USA is obtained through an employer (which caters for the majority), medicaid, medicare and other supplementary insurance programs under the Affordable Care Act (ACA). The rare alternative is self-purchase of health insurance by an individual. However, rural dwellers are more likely to be self-employed or employed by smaller firms. This phenomenon leaves them without employer insurance. Hence those who do not qualify for any of the programs under the ACA risk the likelihood of being uninsured [18]. This is because rural families who lack the employer insurance and are not eligible for any of the ACA programs are compelled to buy individual health insurance policies on their own from a marketplace. This tends to be more expensive compared to policies offered by employers. In general, individual policies disadvantageously provide less comprehensive coverage with high deductibles and co-pays. Consequently, they decide to pursue limited insurance. Also, the high costs within the insurance market have been partly attributed to the ACA, as it allows consumers the flexibility of dropping in and out of insurance markets.

Another peculiar characteristic of rural inhabitants that leads to a high propensity of market failure in the health insurance market is age. According to the demographics report by ERS,^2^ the average age of rural dwellers in 2012 was 58 years, which was much higher than the average national age. Meanwhile, it is open knowledge that health challenges and therefore healthcare costs increase with age [14]. This implies that health insurance issuers become potentially exposed to high losses if they deal in rural areas compared to areas with a lower average age. As a result of the concept of costs in economic theory that “There Ain’t No Such Thing As A Free Lunch”,^3^ the cost must either be borne by the consumers or the health insurance issuers.

The contribution of this paper is two-fold; first we compare the relative impact of pre-existing conditions to risk attitudes using parameters estimated within a principal-agent equilibrium. Secondly, we introduce a set of risk attitudinal indices to examine the behaviour of individuals with respect to health insurance demands. This paper is organized into six sections. In section 2, we present the current state of health insurance in the US and evidence of health insurance demands. In section 3, we present the methodology and data. This section also includes a conceptual framework and the theoretical underpinnings. The results and discussions are presented in section 4. In the penultimate section, we present the conclusions drawn. Finally, policy implications of our results and suggestions are presented in section 6.

## Literature review

In 2010, the Patient Protection and Affordable Care Act (ACA) was enacted. The provisions within this act aimed to expand access to insurance, increase consumer protections, and emphasize prevention and wellness. Other objectives were to improve quality and system performance, expand the health workforce, and curb rising healthcare costs. It also stated the expansion of services at community health centres. These centres provide access to primary and preventive care for about 7.5 million rural Americans. The USDA [31] reported that the ACA had made significant efforts to reduce the anxieties associated with healthcare expenditure among farmers and rural dwellers. Some policies of the ACA listed by the USDA include doubling the size of the National Health Service Corps, offering scholarships and loan repayments to health practitioners as a reward for practicing in rural areas.

Despite this improvement, rural farming communities are perceived to be very risky health insurance markets. The use of chemicals such as pesticides and heavy-duty machinery reduce the safety associated with most rural communities. The Bureau of Labor Statistics in 2015 revealed that the agricultural sector recorded an incidence rate of 5.7 injuries and illnesses per 100 full-time workers. This was 2.4 points greater than the national average for all industries in the United States. These statistics make the provision of health insurance for farmers risky and subsequently lead to an increase in prices. High prices of these insurance products are inclined to attract individuals who are more susceptible to health hazards or illness, with adverse effects on demand.

Several studies have been conducted to identify the factors affecting health insurance demands in various geographical locations. Diverse groups of variables have been used to explain asymmetry of information in these markets. Among the two categories that have dominated adverse selection studies, the existence of chronic or pre-existing conditions has been conspicuous [2, 7]. . Despite the success of using pre-existing conditions, the basic flaw is the assumption of homogeneity of risk^4^ preferences. Chiappori and Salanie [9] stated that risk aversion is extremely heterogenous and a key determinant of insurance demands. Landsberger and Meilijson [17] is one of the nascent studies to account for the heterogeneity of risk preferences. Since then, there has been a proliferation of studies accounting for the heterogeneity of risk preferences [12, 15, 26]. Other studies have also been conducted with a blend of the two [16, 24]. The communal conclusion among these studies is that risk averse individuals are more inclined to purchase insurance compared to individuals who are more risk tolerant. A synthesis of literature showing the group of variables employed are presented in Table 1.

**Table 1:** Selected literature on adverse selection variables

Authors (Year) “Country of study”	Socioeconomic Variables	Adverse Selection Variables
Studies with Pre-existing condition variables		
Cardon and Hardel [7] “US”	Age, Sex, Income, Region,	Self-reported heath state, Health Care Cost
Zhang and Wang [34] “China”	Age, Sex, Marital status, Education, Family size, Income, Type of house	Existing chronic condition
Gao et al. [13] “China”	Age, Education, Sex, Marital Status, Occupation	Wealth
Resende and Zeidan [25] “Brazil”	Sex, Age, Income, Number of dependents and Highest Educational Level	Occurrence of illness
		Occupational Groups
Bolhaar et al. [4] “Ireland”	Age, Sex, Educational Level, Employment Status, Household size, Number of children, Marital status, Habitation, Income, Insurance option from Employer,	GP visits, Specialist visits, Hospital nights, Women that gave birth, Poor mental health, Existing Health Problem, Obese, Daily smoker
Spenkuch [29] Mexico”	Age, Sex, habitation, education, household size, household assets, household expenditure, healthcare expenditure.	Self-rated health status, BMI, Blood pressure, Preventive care, medical utilization
Dardanoni and Donni [11] “United States”	Age, Sex, Education, Wealth, Employment, income	Hospital admission, average number of disease
Studies with variables representing Risk Attitudes		
Schmitz [26] “Germany”		11-point scale on willingness to take risk
Johar and Savage [15] “Australia”	Age, Education, Income, Cognition, Expectation	Risk Tolerance
Studies with variables representing both Pre-existing Conditions and Risk attitudes		
Buchmueller et al. [6] “Australia”	Age, Sex, Income, Highest Educational Level	Risk Attitudes
		Smoker Status, Level of activity (Exercises)
		Checks of Freckles and Moles, Kessler PDS
		Pre-existing Condition
		Inpatient Stay in the past 12 months
		Self-reported health condition
Keane and Stavrunova [16]. “United States”	Age, Sex, Race, Marital Status, Highest Level of Education, Income	Pre-existing Condition
		Health factor, Total medical expenditure
		Subjective probability to live to 75 years or more
		Risk Attitude
		Risk tolerance, Financial planning Horizon
Polyakova [24] “Germany”	Age, Sex, Income, Worktime, Educational Level, Marital Status, Number of Children, House size, Occupation of spouse, Employs house help	Pre-existing Condition
		BMI, annual no. of outpatient visits, inpatient stays, smoker status, Self-reported suffering from diseases (asthma, cancer, cardiac, dementia, depression, diabetes, high blood pressure, migraine, stroke)
		Risk Attitude
		Question on desire to take health risks

### Where does the tree fall?

Information asymmetry in health insurance markets had been measured based on the positive correlation test proposed by Chiappori and Salanie [10]. Their underlying assumption was based on the correlation between an individual’s healthcare utilization (a proxy for pre-existing or chronic conditions) and the demand for insurance. Yet, this framework has drawn quite a few critiques to itself, in that; it does not depend on the market structure or specific properties of preferences. Meanwhile, evidence indicates that the consumers in health insurance markets have heterogeneous observed and unobserved characteristics which may influence the preference for insurance. This flaw makes the Principal Agent (P-A) model a handy alternative framework. The P-A model establishes an equilibrium for insurance preferences while accounting for latent and global incentive compatibility characteristics of the agent. To incorporate risk attitudes in a model to assess adverse selection, the positive correlation analysis falls short in the sense that correlation does not necessarily demystify causality. Furthermore, characteristics such as these are likely to be revealed through incentivization which is in-built in the P-A model. On this bedrock, we employ the P-A model as the analytical framework to assess adverse selection in rural insurance markets.

## Methodology and method of analysis

### Conceptual framework

**Fig. 1:**
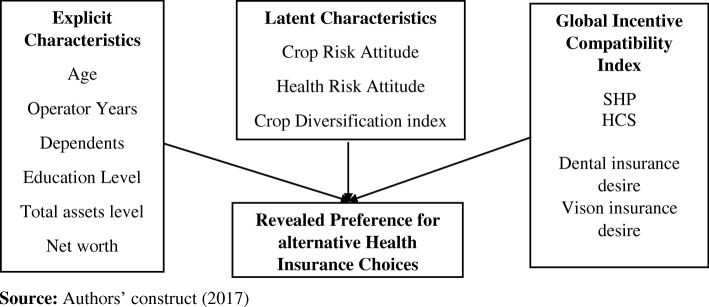
Conceptual Framework for the determinants of Health Insurance

The conceptual framework (Fig. 1) dwells on the idea of the P-A model. In general, equilibrium is established in this model if there is symmetry of information between the principal and the agent. This research focusses on the determinants of the choice of health insurance type by farmers in rural America. Under equilibrium in the P-A model, the agents’ choice is determined by their explicit characteristics, latent characteristics which satisfies the necessary conditions of the model and the global incentive compatibility index. In perspective to this paper, the global incentive compatibility index contains two variables representing pre-existing or chronic conditions; significant health conditions in the past 5 years (SHP) and annual healthcare spending (HCS), while the latent characteristics represent the risk attitudes.

### Theoretical framework^5^

In this paper, the P-A model of Shadnam [28] is expanded to measure adverse selections using factors which were reviewed from literature to affect health insurance demands. It is assumed in this case without any loss of generality that the principal is the insurance company while the agent is the rural dweller. The choice of health insurance is defined by *φ* which envelopes a set of characteristics; these include both explicit (*ω*) and latent (*γ*) characteristics from the perspective of the principal. Among the observable characteristics known by the insurance companies, the most important is the proportion of agents who are willing to obtain the health insurance plan at varying premiums. This and other observable characteristics are classified as *ω* in this model, whereas the unobservable characteristics are denoted by *γ*. In this model, *φ* represents the preference of the agent which belongs to a bounded domain *Θ* ∁ *ℝ*^*P*^. The respondent (agent), who is assumed to have a preference level *φ*, purchases a health insurance product from an insurance company. The utility derived is based on the source and this is represented by ${z}_i\in {\mathbb{R}}_{+}^m$. The agent pays a dollar amount, *d* ∈ *ℝ*_+_, as the insurance premium. Assuming a ceteris paribus condition, the utility of this agent (*U*_*f*_) can be modelled as;

1 $$ {U}_f\left(\varphi \right)=h\left(\varphi, z\left(\varphi \right)\right)-d\left(\varphi \right) $$

It is only an approximation that *U*_*f*_(*φ*) is a quantification of how much an agent, with preference level *φ* enjoys the health insurance product from source *z*_*i*_, knowing that he spends an amount of *d* on it. The administrative and other costs associated with maintaining and fulfilling the obligations of a health insurance product by the principal are given as *C*(*z*_*i*_) of source *z*_*i*_. Therefore, the utility of the principal which is represented by *π* can also be defined as the profit obtained from providing health insurance products *z*_*i*_ to the agent with preference level *φ*. This can be written as;

2 $$ {U}_{\pi}\left(\varphi \right)=d\left(\varphi\ \right)-C\left(z\left(\varphi \right)\right) $$

Since insurance is a risk pooling mechanism, the goal of the insurance company is to maximize profits enough to cover administrative costs and associated indemnities to be paid. The preference for insurance by the agents is based on *φ*. Due to this, its utility *U*_*π*_(*φ*) will be subject to a set of constraints despite efforts to mitigate the probability of having only risk-prone agents in its pool. These constraints are the unobservable (*γ*) characteristics of the agents and as such, there must be an applied effort by the insurance companies to incentivise the agents to reveal these characteristics. These are widely referred to as incentive compatibility constraints. A mathematical representation of the incentive compatibility constraint is;

3 $$ h\left(\gamma, z\left(\gamma \right)\right)-d\left(\gamma \right)\ge h\left(\gamma, z\left({\gamma}^{\prime}\right)\right)-d\left({\gamma}^{\prime}\right)\kern0.5em \forall \gamma, {\gamma}^{\prime}\in \varTheta $$

In this case, the principal-agent problem can be modelled in a discrete choice framework as;

4 $$ \operatorname{Max}\kern0.5em \left({z}_i,{d}_i\right)\kern0.75em {\sum}_{i=1}^n\left({d}_i-C\left({z}_i\right)\right){f}_i $$

$$ \mathrm{s}.\mathrm{t}h\left({\gamma}_i,{\omega}_i,{z}_i\right)-{d}_i\ge 0,\kern0.75em {\forall}_i=1\dots \dots \dots \dots \dots \dots n\ \left(\mathrm{IR}\right) $$

$$ h\left({\gamma}_i,{\omega}_i,{z}_i\right)-{d}_i\ge h\left({\gamma}_i,{\omega}_i,{z}_j\right),\kern0.75em {\forall}_j=1\dots \dots \dots \dots \dots \dots n\ \left(\mathrm{IC}\right) $$

IR is the rationality constraint, which implies that agents will only purchase health insurance if they receive at least a zero-utility level. IC represents the incentive compatibility constraint. The optimal and expectant equilibrium for this P-A model is a Bayesian-Nash Incentive-Compatibility (BNIC). This is an equilibrium in which both the principal and agent must act truthfully and reveal true preferences to obtain the best outcome [27]. The complete information assumption of the BNIC is a limitation in practical insurance. An adverse selection is present when we have incomplete information. In this regard, we introduce the category of agents whose hidden information can affect the equilibrium of the P-A model established in eq. 4.

Following Chavas [8], insurance firms are assumed to be risk neutral whereas the agents, based on their latent characteristics are classified under two groups;

- Group *γ*_*a*_: “low risk” individuals who face a prospect of loss *τ*_*a*_(*e*) > 0,
- Group *γ*_*b*_: “high risk”^6^ individuals who face a prospect of loss *τ*_*b*_(*e*) > 0, *E*(*τ*_*a*_) < *E*(*τ*_*b*_)

Because the IR constraint still holds for the agents, they are assumed to have identical risk-averse preferences, in that, *U*(−*τ*) implies;

- *EU*(−*τ*_*a*_) = *U*(*E*(−*τ*_*a*_) − *R*_*a*_), for individuals in “group A”
- *EU*(−*τ*_*b*_) = *U*(*E*(−*τ*_*b*_) − *R*_*b*_), for individuals in “group B”
- *R*_*a*_ and *R*_*b*_ > 0, are the risk premium.

Under ideal conditions of the BNIC, the insurance companies should know the percentage components of agents in each group; where *α and* (1 − *α*) are assumed for the percentage of individuals in groups *τ*_*a*_ and *τ*_*b*_ respectively. But asymmetry of information makes this situation unattainable for the insurance companies. Hence, insurance companies will offer health insurance contracts for the loss *τ*, with an expected value of premiums being equal to the expected value of loss among all individuals, represented as;

5 $$ \kern0.5em \tau =\alpha E\left({\tau}_a\right)+\left(1-\alpha \right)\Big(E\left({\tau}_b\right) $$

Individuals in group “B” will accept this contract if

6 $$ U\left[-E\left({\tau}_b\right)-{R}_b\right]= EU\left(-{\tau}_b\right)<U\left[-\alpha E\left({\tau}_b\right)-\left(1-\alpha \right)\right(E\left({\tau}_b\right)\Big] $$

However, the group of individuals in “A” will not accept this contract if

7 $$ U\left[-E\left({\tau}_a\right)-{R}_a\right]= EU\left(-{\tau}_a\right)>U\left[-\alpha E\left({\tau}_a\right)-\left(1-\alpha \right)\right(E\left({\tau}_b\right)\Big] $$

Due to this self-selection behaviour of low risk individuals, the insurance companies become vulnerable to high anticipated losses and therefore an equilibrium contract is not feasible. This can lead to market failure if not solved. Meanwhile, the Spence-Mirrlees Condition (SMC) must be met for the solution of this problem to be obtained. According to Araujo and Moreira [1], the strength of the SMC for hidden information problems is to provide a full characterization of implementable contracts using only the local incentive compatibility (IC) constraints. In this paper, these constraints are equivalent to the monotonicity of the decision variable with respect to the agent’s latent parameter (*γ*). They further emphasize that a violation of the SMC makes the local IC constraints no longer sufficient for implementation and therefore additional (global) IC constraints must be considered. This SMC is defined by Shadnam [28] as the marginal rate of substitution between the quality of the health insurance product and premium paid (either increasing or decreasing with the agent’s preference). The SMC condition is not satisfied in many utility functions leading to market failure, hence we introduce the unobservable characteristics of the comparative statics in the empirical section.

### Empirical model specification

The empirical model formulation and statement of hypothesis is well grounded in the conceptual and theoretical framework in preceding sections. To establish the empirical model, we obtain the first order conditions from the constrained maximization problem in eq. 4;

8 $$ \ell \left({\gamma}_i,{\omega}_i,{\lambda}_0,{\lambda}_1\right)={\sum}_{i=1}^n\left({d}_i\left({\gamma}_i,{\omega}_i\right)-C\left({z}_i\Big({\gamma}_i,{\omega}_i\right)\right){f}_i\Big)+{\lambda}_0\left[\left(h\left({\gamma}_i,{\omega}_i,{z}_i\Big({\gamma}_i,{\omega}_i\right)-{d}_i\left({\gamma}_i,{\omega}_i\right)\right)\right]-\kern0.5em {\lambda}_1\left({d}_i\left({\gamma}_i,{\omega}_i\right)\right) $$

The partial derivatives of equation (8) with respect to *γ*_*i*_, *ω*_*i*_, *λ*_0_ *and λ*_1_ are given as;

9 $$ {\ell}_{\lambda_0}=\left(h\left({\gamma}_i,{\omega}_i,{z}_i\Big({\gamma}_i,{\omega}_i\right)-{d}_i\left({\gamma}_i,{\omega}_i\right)\right) $$

10 $$ {\ell}_{\lambda_1}=\left({d}_i\left({\gamma}_i,{\omega}_i\right)\right) $$

11 $$ {\ell}_{\gamma_i}=\frac{\partial {\sum}_{i=1}^n(.)}{\partial {\gamma}_i}-\frac{\partial C(.)}{\partial {\gamma}_i}+\frac{\lambda_0\partial h(.)}{\partial {\gamma}_i}-\frac{\partial {d}_i(.)}{\partial {\gamma}_i}-\frac{\lambda_1\partial {d}_i(.)}{\partial {\gamma}_{\mathrm{i}}} $$

12 $$ {\ell}_{\omega_i}=\frac{\partial {\sum}_{i=1}^n(.)}{\partial {\omega}_i}-\frac{\partial C(.)}{\partial {\omega}_i}+\frac{\lambda_0\partial h(.)}{\partial {\omega}_i}-\frac{\partial {d}_i(.)}{\partial {\omega}_i}-\frac{\lambda_1\partial {d}_i(.)}{\partial {\omega}_i} $$

Using comparative statics, equations (8-12) lead to optimal equilibrium solutions *λ*_0_^∗^, *λ*_1_^∗^, *γ*_*i*_^∗^ *and ω*_*i*_^∗^, of which *λ*_0_^∗^ and *λ*_1_^∗^ are regarded as redundant. Hence *γ*_*i*_^∗^ and *ω*_*i*_^∗^ are the necessary solutions for the maxima. Considering the discrete nature of the health insurance alternatives,^7^ a link function model^8^ is appropriate for the estimation. The multinomial logit and complementary log-log binomial link function are employed as the estimation methods. The multinomial logit model is traditionally used because the alternatives are more than two. It imposes the assumption that the respondent is the unit of analysis and hence, based on the respondent’s characteristics. This is given as;

13 $$ {P}_{ij}=\frac{\exp \left({\Delta }_k^{\prime }{X}_j\right)}{\sum_{l=1}^m\exp \left({\Delta }_i^{\prime }{X}_j\right)} $$

Where ${\Delta }_1^{\prime}\dots {\Delta }_m^{\prime }$ represents *δ*_*i*_, *β*_*i*_ *and ρ*_*i*_ which are vectors of unknown regression parameters of the variables (*X*) in the P-A equilibrium.

From Table 4, the choice of insurance by the respondents is highly skewed towards having insurance. The complementary log-log binomial link function is a generalized linear model that allows for an asymmetric dependent variable. It is also beneficial in cases of potential confounding or effect modifiers [22]. This is given as;

14 $$ \mathit{\log}\left(-\log \left(1-{p}_i\right)\right)=\log \left({u}_i\right)+\mathit{\log}\left({A}_i\right) $$

Where *u*_*i*_ is the unknown parameter, *p*_*i*_ is the probability of choosing insurance and *A*_*i*_ is the offsetting term.

Expanding the multinomial logit or the complementary log-log binomial (CLL) models and presenting them empirically gives;

15 $$ {P}_{ij}={\delta}_0+{\delta}_1 Opyrs+{\delta}_2 Depend+{\delta}_3 LnNW+{\delta}_4L\mathrm{n} TA+{\delta}_5 EDU+{\delta}_6 Age+{\beta}_1 LnHCS+{\beta}_2 SHP+{\beta}_3 DENCOV+{\beta}_4 VISCOV+{\rho}_1 Healthriskatt+{\rho}_2 Cropriskatt+{\rho}_3 HHIndex $$

The null hypothesis is specified as;

16 $$ {H}_0:{\beta}_i;{\rho}_i>0 $$

Implying that, the coefficients of the latent and global incentive compatibility variables will be positive in the presence of minimal information about the risk prospects of the agent revealed to the principal. This implies that the probability of health insurance demands will be increasing (Table 2).

**Table 2:** Summary of symbols used for variables in equations

Symbol	Meaning	Symbol	Meaning
Principal-Agent Model		Crop diversification Index	
*φ*	Health Insurance preferred	*W*_0_	Initial Wealth
*ω*	Explicit characteristics	*q*_*n +* 1_	Terminal Wealth
*γ*	Latent Characteristics	*q*_*i*_	Output from cropped area i
*U*_*f*_	Utility of farmer (agent)	*q*_*j*_	Output from cropped area j
*C*(*z*_*i*_)	Administrative costs of Health insurance companies	*E*(*y*)	Expected Returns from initial wealth
*π*	Utility of Health Insurance Company	*r*	Expected net return on the *ith* asset
IC	Incentive compatibility constraints	*⋋*	Risk Attitude
IR	Rationality constraints	*σ* ^2^	Variance of Portfolio
*γ*_*a*_	“Low risk” Individuals	*y*_*CE*_	Certainty Equivalent
*γ*_*b*_	“High risk” Individuals	*s*_*i*_	Optimal share of crop
*τ*	Health insurance loss for unknown proportions	*A*_*i*_	Area of available farming land used for crop i
*τ*_*a*_(*e*)	Loss prospect for *γ*_*a*_	n	Number of crop portfolio choices
*τ*_*b*_(*e*)	Loss prospect for *γ*_*b*_	*ρ*_*τ*_	Tau-Equivalent
*E*(*τ*_*a*_)	Expected loss prospect for *γ*_*a*_	Test for IIA	
*E*(*τ*_*b*_)	Expected loss prospect for *γ*_*b*_	*B*	First Regression
*R*_*a*_	Risk premium for *γ*_*a*_	*b*	Second Regression
*R*_*b*_	Risk premium for *γ*_*b*_	*β*_*B*_	Coefficient of *B*
*α*	% of respondents in *τ*_*a*_	*β*_*b*_	Coefficient of *b*
1 *− α*	% of respondents in *τ*_*b*_	*∂*_*B*_	Covariance of *B*
*m*_*p*_	Alternative profile of options for health risk management	*∂*_*b*_	Covariance of *b*
*f*_*i*_	Probability density function for consumer preference	Multinomial Logit Regression	
Tau Equivalent Test			
X	Number of scale statements	*δ*_*i*_	Coefficient of explicit variables
${\sigma}_{Y_i}^2$	variance of the scores of each scaled statement	*β*_*i*_	Coefficient of global compatibility constraints
${\sigma}_X^2$	the total variance of scores on the respondents’ scales	*ρ*_*i*_	Coefficient of latent variables
Diverse states of nature health insurance decisions by farmers			
	*p*_1_(*s*_1_)		*p*_2_ (*s*_2_)
	Good health		Bad health
*Z*_0_ (no health insurance)	W + *W*_0_		W + *W*_0_ - H
*Z*_1_ (private health insurance)	W + *W*_0_ - *π*_*p*_		W + *W*_0_ - *π*_*h*_ - *D*_*h*_ + *R*_*p*_
*Z*_2_ (government insurance plan)	W + *W*_0_ - *π*_*m*_		W + *W*_0_ - *π*_*m*_ - *D*_*h*_ + *R*_*m*_

### Data sources and variables used in the analysis

The data used for this study is secondary data obtained from North Dakota and Minnesota farm business management education program participants. This group are members of marketing clubs who have reliably provided production and financial data for policy benchmarking since 1994 on annual basis. This data contained 774 observations. Relevant variables for this study were used. The estimation models incorporated three groups of variables as established from the P-A model. The first group are the explicit variables age, experience, number of dependents, net worth and total assets of the agent. The remaining two groups are the adverse selection proxies. The latent characteristics (risk attitudes) are the health risk attitude, crop risk attitude and crop diversification index (Herfindahl-Hirschman index); while the global compatibility (chronic diseases or pre-existing conditions) variables are significant health conditions in the past 5 years and annual healthcare spending.

The development of the risk attitudes are in two folds; (i) based on their crop production decisions and (ii) based on their health perceptions. Six (6) Likert scale statements were used for the crop risk attitudinal scale while fifteen (15) statements were used for health risk attitudinal scale. Both categories of scale had a 10-point Likert response in which farmers indicated their extent of agreement with 1 representing total agreement and 10 representing total disagreement. In general, health insurance issuers price insurance policies based on the age, sex, smoking status and state of residence. Hence it would have been ideal to have all these variables included in the model. However, the limitation of the dataset is that smoking status was not collected. Furthermore, the incorporation of state of residence may not yield any policy viable impact considering that the respondents were drawn from only North Dakota and Minnesota. The list of variables, descriptions, dimensions and “a priori” expectations can be found in Table 3.

**Table 3:** Variables used, their description, dimensions and “a priori” expectations

Variable	Description	Dimension	“a priori” expectation
Explicit Characteristics			
Opyrs	Number of Years Operator has been farming	Absolute number of years	Positive
Age	Age of operator	Absolute number of years	Positive
Depend	Number of dependents of the operator	Absolute number of years	Positive
LnNW	Log of net worth of operator	Index	Negative
LnTA	Log of total assets of operator	Index	Negative
Edu	Highest educational level of operator	Base group: High School	Positive
Some College	If operator’s highest level of education is some college degree	Dummy (1 if yes, 0 if otherwise)	Positive
College Grad and above	If operator’s highest level of education is College Graduate	Dummy (1 if yes, 0 if otherwise)	Positive
Global Incentive Compatibility Index (Other variables)			
Dencov	If operator needs a dental plan as part of a health insurance plan	Likert response (1 for strongly agree; 10 for strongly disagree)	Positive
Viscov	If operator needs a dental plan as part of a health insurance plan	Likert response (1 for strongly agree; 10 for strongly disagree)	Positive
Global Incentive Compatibility Index (Proxies for pre-existing or chronic conditions)			
LnHCS	Log of annual health care costs of operator	Index	Test Variable
SHP	Significant health Conditions in the past 5 years	Dummy response (0 for Yes and 1 No)	Test Variable
Latent Characteristics (Proxies for Risk Attitudes)			
Healthriskatt	Risk Attitude based on Health Likert scale statements	Average from Likert response	Test Variable
Cropriskatt	Risk Attitude based on Crop Likert scale statements	Average from Likert response	Test Variable
HHIndex	Herfindahl Coefficient of Diversification	0: least diversified	Test Variable
		1: Highly diversified	

## Results and discussions

### Descriptive statistics

The summary statistics of key variables can be found in Table 4. In this revealed preference analysis, it was found that 96.51% of the respondents had insurance. In terms of the sources, 5.81% were from government sources while 90.70% were from private insurance sources. The mean age of the respondents was found to be 45.98 years with a minimum of 23 and maximum of 76 years. The years of farming experience of the respondents ranged from 1 to 65 years with a mean of 25.35.

**Table 4:** Moments of selected variables

Variable		Mean	Std. Dev	Minimum	Maximum
Age (Years)		45.98	11.06	23	76
Experience of Operator (Years)		25.35	16.39	1	65
Total Assets (US$)		1155233	1290349	0	6000000
Net Worth (US$)		674186	765618	0	2700000
Annual Health Care Spending (US$)		5627.91	3442.06	0	11000
Health Insurance	Percentage	Health Insurance Source			Percentage
Insurance	96.51	Government insurance			5.81
		Private insurance			90.70
No Insurance	3.49	No Insurance			3.49
Operator Age (Years)	Percentage	Operator Experience (Years)			Percentage
21–40	27.91	0–20			36.05
41–60	59	21–40			52.33
Over 61	10.47	41–60			8.14
Highest Education	Percentage	61–80			3.49
High School	18.60	Total Assets (US$)			Percentage
Some College	33.72	0–1,499,999			62.79
College Graduate and above	47.67	1500,00 0–2,999,999			25.58
Net Worth (US$)	Percentage	3000,00 0–4,499,999			0
0–499,999	62.79	4,500,000 -5,999,999			8.14
500,000 -1,499,999	17.44	Over 6,000,000			3.49
1500,000 -2,499,999	13.95				
2,500,000	5.81				

The Cronbach alpha values for these were 0.5226 and 0.6807 for the crop and health risk attitudes respectively. Despite these values falling short of the 0.7 which is the rule of thumb for acceptance of international consistency, White et al. [33] justified the validity of alpha values above 0.5. Therefore, these values were accepted in this paper. (Results can be seen in Table 5).

**Table 5:** Cronbach alpha values of likert scale statements for crop risk attitudes

Statement Crop Risk Attitudes		Correlation with Total	Alpha if Item Deleted
Crop insurance is a safety net that should only pay in times of disaster		0.1457	0.5371
Availability of high coverage levels (> 75%) is important to me		0.2717	0.4769
Per-acre premium costs are very important to my crop insurance decisions		0.5509	0.3259
I choose a crop insurance product that will return the most indemnity payments per premium dollar paid		0.5410	0.3317
I select whatever crop insurance product my agents recommend		0.3078	0.4587
Crop insurance is too complicated to understand and use		− 0.0889	0.6371
Overall Cronbach Alpha Coefficient Value			0.5226
Health Risk Attitudes			
Health insurance is not important to my farm operation		0.1869	0.6791
I should get a return on my investment when I buy health insurance		0.1968	0.6778
Deductible levels are important to my health insurance purchase decisions		0.1913	0.6786
I would be more willing to hold health insurance if I had access to large risk groups		0.3431	0.6579
Prescription drug benefits are important to me when choosing health insurance		0.4279	0.6459
I need dental coverage as part of my health insurance package		0.2094	0.6761
I need vision as part of my health insurance package		0.4493	0.6428
Health insurance is more expensive than for farmers than other occupations		0.3360	0.6589
I think health insurance is necessary to protect my farm operation		0.2251	0.6740
Farmers should have risk groups similar to employer- based insurance risk groups		0.4739	0.6392
Large pool farmer risk groups should be mandated by government and implemented by private industry		0.4784	0.6386
Large pool farmer risk groups should be mandated and implemented through government programs		0.4404	0.6441
A subsidy that blends crop and health insurance would manage farm risk better than just crop insurance		0.2353	0.6727
A program that blends crop and health insurance is not important to my farm operation		−0.0008	0.7033
Overall Cronbach Alpha Coefficient			0.6807
Domain		Crop Risk Attitude	Health Risk Attitude
Results of Risk Attitudes of respondents			
Risk Loving	Frequency	117	153
	Percentage	(15.12)	(19.77)
Risk Neutral	Frequency	0	9
	Percentage	0	(1.16)
Risk Averse	Frequency	657	612
	Percentage	(84.88)	(79.07)
Total	Frequency	774	774
	Percentage	(100)	(100)

### Results of the test for Independence of irrelevant alternatives

The Hausman and McFadden test revealed a validity of the assumption of independence of irrelevant alternatives. The null hypothesis is that the difference in coefficients is not systematic. From the results shown in Table 6, the null hypothesis cannot be rejected and hence we conclude that the difference in the coefficients for the two set of regressions is not systematic. The results imply that there is no correlation in unobserved factors over the choices and hence the use of the multinomial logit is justified.

**Table 6:** Results of hausman test for independence of irrelevant alternatives

	Coefficients			
	B	(B) All Categories	b- (B) difference	Sqrt (diag (V_b -V_B)) S.E.
Years of experience	−0.0292	− 0.0277	− 0.0015	0.0044
Age of operator	0.0120	0.0247	−0.0047	0.0089
Number of dependents	2.2541	−0.2038	1.3326	1.0852
Net worth	−1.3334	−0.4270	−0.9064	0.7207
Annual healthcare cost	−4.9046	−2.4750	− 2.4296	2.0015
Total Assets	0.7113	0.3697	0.3416	0.5194
Desire for dental coverage	−3.1500	−1.6047	−1.5453	1.3602
Desire for vision coverage	−0.0746	−0.0946	−0.1692	0.1513
Health risk attitude	0.1899	0.0479	0.1420	0.0741
Crop risk attitude	0.2560	0.2379	0.0181	0.0449
Herfindahl-Hirschman Index	4.3067	3.8538	0.4530	0.9069
Some College	−13.1221	−5.9414	−7.1807	5.5785
College Graduate	−17.1068	−7.8900	−9.2168	7.0984
Significant Health Condition in past 5 years	32.7062	22.6988	10.0074	1490.9540
Constant	18.8889	−1.9743	20.8633	1491.017
Chi (5) = 1.44 Prob >chi2 = 0.9199	Null hypothesis: Difference in coefficients is not systematic			

b = consistent under Ho and Ha; obtained from multinomial logit

B = inconsistent under Ha, efficient under Ho; obtained from multinomial logit

## Multinomial logit results

The software used for the analysis of this study is the Stata version 14 software. The multinomial logit regression (Table 7) shows that the factors affecting the decision on government health insurance are years of farming experience, number of dependents, net worth, total assets, annual health cost spending, significant health problems in the past 5 years, highest educational level attained, the extent of preference for vision coverage in a health insurance package and the Crop Diversification Index. The base group for this regression is the group that had no insurance. The regression has a log likelihood of − 161.87 and is significant at a chi-square percentage probability of 1% while the Pseudo R-Squared is 43.63%.

**Table 7:** Factors affecting the selection of health insurance among farmers from multinomial logit model

Dependent Variable: Health Insurance Preference		Coefficient (Standard Errors)		Marginal Effects		
Base Group: No Insurance Holders		Private insurance	Government insurance	No Insurance	Private Insurance	Government Insurance
Years of Experience		−0.277*	0.035*	0.0006	−0.0006	0.0003
		(0.017)	(0.019)	(0.00038)	(0.00055)	(0.0004)
Age of Operator		0.025	−0.0298	0.0005	−0.0003	− 0.00022
		(0.019)	(0.025)	(0.0005)	(0.0008)	(0.00067)
Number of Dependents		−0.922***	−1.881***	0.0206	0.018	−0.039
		(0.132)	(0.265)	(0.0085)	(0.011)	(0.0083)
Net Worth of Operator		0.427**	0.535***	−0.009	0.0047	0.0046
		(0.207)	(0.208)	(0.0042)	(0.0043)	(0.0012)
Annual Health Care Spending by Operator		2.475***	3.538 ***	−0.0542	0.0104	0.0438
		(0.215)	(0.444)	(0.0069)	(0.015)	(0.014)
Total assets of Operator		−0.3697*	− 0.822***	0.0083	0.0098	−0.0182
		(0.193)	(0.198)	(0.0040)	(0.0044)	(0.014)
Highest Educational Level						
Some College		5.94***	5.419***	−0.1673	0.0364	−0.0004
		(0.570)	(0.678)	(0.0165)	(0.0226)	(0.0188)
College Graduate and above		7.889***	−7.687***	−0.1903	0.1935	−0.0032
		(0.957)	(1.028)	(0.0181)	(0.0238)	(0.0159)
Significant Health Condition in Past 5 years		−22.698***	−25.335***	0.04832	0.07548	−0.1238
		(1.165)	(1.239)	(0.0057)	(0.0245)	(0.0238)
Desire for Dental Insurance Coverage		1.60***	1.656***	−0.0347	−0.0315	− 0.0031
		(0.180)	(0.181)	(0.0049)	(0.0048)	(0.0006)
Desire for Vision Insurance Coverage		−0.095	0.059	0.0019	−0.0079	0.006
		(0.074)	(0.097)	(0.0017)	(0.0033)	(0.0053)
Health risk attitudes		−0.048	0.086	0.00094	−0.006	0.0052
		(0.185)	(0.234)	(0.004)	(0.0069)	(0.0058)
Crop risk attitudes		0.238	−0.418	0.0050	0.002	−0.007
		(0.278)	(0.307)	(0.005)	(0.0078)	(0.0053)
Herfindahl-Hirschman Index		−3.854	−4.336	0.0836	0.0617	−0.0218
		(2.510)	(3.009)	(0.0503)	(0.073)	(0.056)
_cons		−1.97	− 3.211			
		(2.699)	(4.470) 1266.01			
Pseudo R-square	0.4363	Wald Chi2(28)				
Number of Observations	774	Prob> Chi2	0.0000			

***, ** and * represents the 1, 5 and 10% significance levels respectively

### Impact of explicit characteristics on insurance choice

The years of farming experience had a positive effect on the choice of private and government insurance compared to the base group. A year increase in the experience of the respondent revealed a 0.02% increase in the probability of desiring private insurance. Among the group of respondents who purchased government insurance, this variable had a 0.03% positive effect on the choice of insurance. However, for the group who had no insurance, a year increase in farming experience decreased the desire to purchase insurance by 0.06%. The number of dependents was found to affect the choice of government insurance negatively with reference to the base group at a significance level of 1%. The marginal effect of − 0.038 revealed that a unit increase in the number of dependents reduced the desire to purchase government insurance by 3.8%. The marginal effect of this variable on the choice of private insurance choice revealed a 1.8% positive influence. For those who did not have any health insurance, a positive marginal effect of 2.0% of the number of dependents was obtained.

Education was found to be a significant determinant of health insurance choice at 1% significance level. In general, an increase in the level of education attained increased their desire for health insurance. However, it was realised that the more educated respondents were more inclined to purchase private insurance than government insurance. Among insurers who had private insurance, having some college education increased the odds of purchasing insurance by 3.64% while having graduated from college or possessing a higher degree increased the odds of purchasing private insurance by 19.35%. For respondents who had no insurance, having some college education reduced the odds of not having insurance by 16.73% while completing a college degree or higher reduced the probability of not being insured by 19.03%. For respondents who reported having government health insurance, the marginal analysis revealed that having some college education reduced the odds of having insurance by 0.04%. On the other hand, having a college education or higher reduced the probability of having government insurance by 0.3%. This depicts the tendency of the higher educated respondents to be concurrently employed along with their farming enterprise and hence, gaining other forms of insurance other than the government insurance.

The net worth of the respondents was found to be significant at 5% for the choice of private insurance and 1% for the choice of government insurance. An increase in the net worth of a respondent increased the probability of purchasing both private and government health insurance by 0.4%. It is significant to note that for respondents who did not have any form of health insurance, an increase in net worth reduced the probability of not having insurance by 0.9%. The total assets possessed by respondents was found to be significant at 10% and 1% for the choice of private and government insurance respectively. The marginal effect of total assets on the desire for private insurance was 0.9% while the desire for government insurance was influenced negatively by a margin of 1.8%. For those who had no insurance, an increase in total assets increased the probability of not insuring by 0.8%.

### Impact of global incentive compatibility index

From the results obtained, the two proxies of pre-existing conditions were significant at 1% for both choices of insurance. An increase in the annual healthcare spending increased the probability of choosing private insurance by 1% while it increased that of government health insurance by 4%. An increase in annual healthcare spending revealed a 5% reduction in the probability of not purchasing health insurance. Respondents who had no significant health conditions in the past 5 years were 22 times and 25 times less likely to adopt private and government insurance than the base group respectively. Among those who had government insurance, having encountered a significant health condition in the past 5 years decreased the probability of having insurance by 12.38%. Studies that also found pre-existing conditions to be a significant determinant of health insurance include Resende and Zeidan [25]; Bolhaar et al. [4]; Dardanoni and Donni [11] and Buchmueller et al. [6]. The results from this section signify the existence of adverse selections in the US rural health insurance markets based on pre-existing conditions.

### Impact of latent characteristics on the choice of health insurance

Considering the prohibition of health insurers from refusing coverage based on patients’ medical history, we examine the impact of these risk attitudes as indicators of adverse selection in this market within the same framework. The variables of interest to this section are the health risk attitude, crop risk attitude and crop diversification index (Herfindahl-Hirschman index). These three variables representing the respondent’s risk tolerance did not play a role in the choice of any of the health insurance options. Similar results were obtained by Landsberger and Meilijson [17], Cardon and Handel [7] and Bajari et al. [2].

### Impact of other variables on choice of health insurance

The desire for dental coverage as part of an insurance plan is found to be significant at 1%. For respondents who possessed private insurance, an increasing desire for dental coverage meant a 3.2% probability of having insurance while a 0.3% probability exists for those who purchased government insurance. An increasing desire for dental insurance reduced the probability of not having insurance by 3.5%.

## Complementary log-log binomial link results (CLL)

The results from this model are largely consistent with that from the multinomial logit model. Considering the asymmetric nature of the dependent variable, the CLL which is well adapted to such data is also employed for robustness of the model. The dependent variable in this model considers not having insurance as one option while having either government or private insurance is considered as one option. From the model, years of experience, age of operator, number of dependents and educational level are significant at 1%. The two variables incorporated to measure pre-existing conditions i.e. annual health care spending of the operator and significant health condition in the past 5 years are significant at 1%. With a base group of yes, the negative coefficient implies that those who had no significant health conditions were less likely to choose health insurance. Other variables that significantly affect the decision to purchase health insurance were 1% for dental insurance coverage and vision insurance coverage. The net worth of the operator affects the choice of health insurance at a significant level of 5%. This variable had a negative coefficient implying that respondents with a higher value of net worth were less likely to purchase health insurance. The three variables used to measure risk attitudes were again insignificant in determining the choice of health insurance. This is similar to the observation from the multinomial logit (Table 8).

**Table 8:** Factors affecting the selection of health insurance among farmers from Complementary log-log binomial model

Dependent Variable (Health Insurance Choice)	Coefficient (Std Error)
Years of Experience	0.001***
	(0.000)
Age of Operator	0.003***
	(0.001)
Number of Dependents	0.016***
	(0.006)
Net Worth of Operator	−0.003**
	(0.002)
Total assets of Operator	−0.001
	(0.002)
Annual Health Care Spending by Operator	0.051***
	(0.012)
Highest Educational Level	
Some College	0.045
	(0.029)
College and above	0.120***
	(0.032)
Significant Health Condition in Past 5 years	−0.075***
	(0.015)
Desire for Dental Insurance Coverage	0.003***
	(0.001)
Desire for Vision Insurance Coverage	0.009***
	(0.003)
Health risk attitudes	0.002
	(0.006)
Crop risk attitudes	−0.01
	(0.007)
Herfindahl-Hirschman Index	−0.100
	(0.069)
Constant	−0.986***
	(0.134)
Log-Likelihood function	−550.166
Number of Observations	774
AIC	1.4604
BIC	− 4983.7730

## Conclusion

Asymmetry of information has been an endemic issue in various contracts including insurance. It generally occurs as either adverse selection or moral hazard. Adverse selection, being the focus of our study, occurs when a party in the contract agreement makes a decision based on a set of information which is not available to the other party. The health insurance markets in the USA have been susceptible to this phenomenon at the detriment of insurance companies. In response, a majority of these companies have pulled out from areas of perceived information asymmetry which encompasses vast rural areas. The measurement of adverse selection in these markets has mainly dwelt on the existence of pre-existing health conditions or closely related proxies. To worsen the information asymmetry challenge of the health insurance markets, insurance issuers were prohibited from refusing coverage based on patients’ medical history. This made it even more difficult for issuers to understand the extent of risk associated with the pool of customers. These sequences have negatively affected farmers who have been prejudiciously categorized within the high health risk group. However, the law on pre-existing conditions was repealed in 2018 raising further debate as to whether the government cares about the sick or not. To understand and explain the information asymmetry problem in these markets, this study poses and answers two questions, (i) is there evidence of adverse selection in rural US health insurance markets if pre-existing conditions are considered as the basis? (ii) does the respondents’ domain specific risk attitude have similar selection effects as pre-existing conditions?

To this cause, we established an optimal equilibrium via the P-A model to test for adverse selections using respondents from rural North Dakota and Minnesota. From the results of the multinomial logit model, we found that the variables representing pre-existing conditions provided strong evidence of adverse selections among rural farmers in the US. On the other hand, the risk attitudes did not have an impact on the choice of the health insurance products. This presents a mixed evidence of adverse selections for stakeholders in general and health insurance issuers. Further studies are recommended to help understand the current phenomenon in rural areas and will be beneficial if directed towards the effects of the characteristics of the ACA plan on its choice in rural areas.

In summary, we conclude that there are very minor or no discrepancies in the choice of alternative health insurance schemes. The variables that affect the choice of private health insurance in rural America are the same for those that determine government health insurance choices. However, the effect of pre-existing conditions on the choice of insurance is not the same as the effect of risk attitudes. For both sources of insurance, the use of pre-existing conditions reveals evidence of adverse selections. On the other hand, the use of domain specific risk attitudes did not reveal the existence of adverse selections in both health insurance products.

## Policy implications and suggestions

The results and conclusions imply that adverse selection exists in rural health insurance markets in the US. However, the law on pre-existing conditions makes it difficult for insurers to understand the riskiness of agents. The use of the health risk attitudes, crop risk attitudes and diversification index reveal no evidence of adverse selection. The major policy implication from this study is that respondents who have pre-existing/chronic conditions tend to patronise health insurance with a higher probability than other counterparts and therefore insurers are likely to incur losses given the law on pre-existing conditions as private information. The 2018 law on the exclusion of individuals with pre-existing conditions may be beneficial to the insurance companies at the expense of the populace. We postulate that this phenomenon will inevitably lead to market failure if not addressed. Meanwhile, the respondents’ risk attitudes, being an alternative indicator of adverse selections as shown in the reviewed literature did not provide any realistic evidence of adverse selections among rural dwellers.

In as much as the protection of rural dwellers is paramount in the establishment of the law on pre-existing conditions, the neglect of its effect on the insurer will subsequently lead to a collapse of the market at the detriment of the agents the policy intended to protect. However, the repeal of this law may lead to most Americans with pre-existing conditions losing the privilege of affordable medical care. We therefore suggest that incentive mechanisms to promote larger risk pools should be reconsidered. In that, even within a single state, expected loss ratio from agents vary, and baring the evidence of adverse selections in this study, the insurers are likely to incur high losses from claims if they deal with limited or no risk pools.

This study focuses on consumer behaviour. However, there is room to analyse the full-fledged relationship between the supply side and demand side. Further studies are also suggested into the effects of competition and strategic interaction among the insurers or principals. Finally, since this study was conducted using respondents from parts of North Dakota and Minnesota, we recommend that similar studies should be conducted in other parts of the country given that demographic characteristics vary across the country. Given the disparity between urban and rural uninsurance levels, an urban analysis is also encouraged to identify the possible lessons drawn to help improve the US rural health insurance markets.

## Data Availability

Data was obtained from the Department of Agribusiness and Applied Economics (NDSU). Interested researchers may contact William Nganje (E-mail: william.nganje@ndsus.edu) for data queries.

## Footnotes

1 Rural uninsured populations could benefit considerably from the health insurance options available on the marketplace. However, ACA’s ability to significantly increase coverage in rural America is dependent upon effective consumer assistance [20]

2 https://www.ers.usda.gov/topics/farm-economy/beginning-disadvantaged-farmers/beginning-farmers-and-age-distribution-of-farmers/A distribution of their demographics can be found on the preceding link.

3 “There Ain’t No Such Thing As A Free Lunch”. This is a pervasive concept in economic theory emanating from the fact that the consumption of scare resources in a capital market society draws competition from others who also want to partake in its consumption. Hence, the providers of that good or service would have to attach some type of cost to it. As explained earlier, provision of health insurance in rural areas with older populations who incur higher healthcare costs due to frequency of illnesses associated with age will provoke higher costs from health insurance issuers. The only other gloomy options left for them are either to stay with such high potential losses and go bankrupt or opt out of these rural areas, with the latter being more evident in the current situation.

4 Page 65 paragraph 2 of Keane and Stavrunova [16] argued for the essence of risk attitudinal variables in the measurement of information asymmetry.

5 For ease of understanding equations in this section, a summary of the notations and their meanings are provided in Table 2.

6 High risk individuals in this framework are those who have chronic conditions.

7 The health insurance alternatives are (i) No health insurance, (ii) Government health insurance and (iii) Private health insurance. These are represented as a function of the attributes. The discrete states of nature and choices are shown in Table 2.

8 These models allow the dependent variable to have a non-normal distribution. Examples include logit, the complementary log-log model and the family of general linear models.
